# Mitral Valve Infective Endocarditis due to Streptococcus pyogenes: A Case Report

**DOI:** 10.7759/cureus.4461

**Published:** 2019-04-15

**Authors:** Cristina Sarda, Giulia Magrini, Sfefano Pelenghi, Annalisa Turco, Elena Seminari

**Affiliations:** 1 Infectious Diseases, Fondazione IRCCS Policlinico San Matteo, Pavia, ITA; 2 Cardiology, Fondazione IRCCS Policlinico San Matteo, Pavia , ITA; 3 Cardiac Surgery, Fondazione IRCCS Policlinico San Matteo, Pavia, ITA; 4 Cardiology, Fondazione IRCCS Policlinico San Matteo, Pavia, ITA; 5 Infectious Diseses, Fondazione IRCCS Policlinico San Matteo, Pavia, ITA

**Keywords:** infective endocarditis, streptococcus pyogenes

## Abstract

Infective endocarditis (IE) due to group A β-hemolytic streptococcus (Streptococcus pyogenes) has rarely been reported in the literature. We herein report a Streptococcus pyogenes native mitral valve endocarditis in a young patient and a review of the literature. The patient had a native mitral valve endocarditis with vegetation; his hemodynamic stability and a short course of antibiotic treatment prevented urgent surgery on the mitral valve. He was previously treated with cefixime and azithromycin for four days and then, upon hospital admission, with vancomycin plus amoxicillin-clavulanate. After the diagnosis of IE due to Streptococcus pyogenes, treatment with gentamicin (3 mg/kg daily) and ampicillin (12 g/day) was implemented. The patient underwent weekly echocardiographic evaluations during antibiotic treatment to document the resolution of the vegetations. He was discharged to home in good clinical conditions after a four-week course of antibiotic treatment.

## Introduction

Infective endocarditis (IE) due to group A, B, C, or G streptococci, including the Streptococcus anginosus group (S.constellatus, S. anginosus, and S. intermedius) is relatively rare [1]. IE due to group A β-hemolytic streptococcus (Streptococcus pyogenes) has rarely been reported in the literature. We herein report a Streptcoccus pyogenes native mitral valve endocarditis in a young patient along with a review of the literature.

## Case presentation

**Figure 1:**
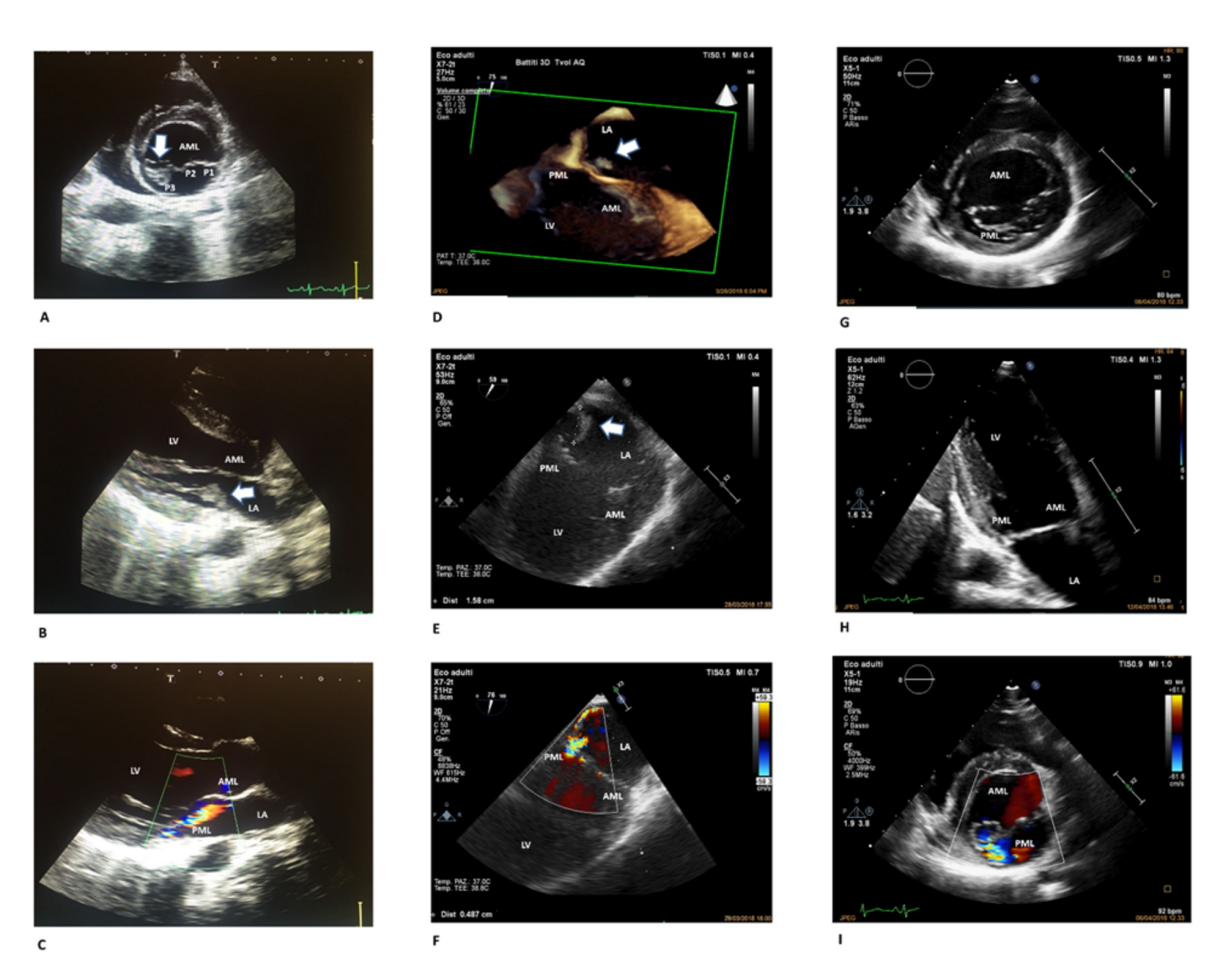
Sequential echocardiographic assessments Panel A: Transthoracic two-dimensional echocardiography – parasternal short axis view of the mitral valve. Arrow showing vegetation on the medial portion of the posterior mitral leaflet (P3). Panel B: Transthoracic two-dimensional echocardiography – parasternal long axis view. Arrow showing vegetation on the posterior mitral leaflet. Panel C: Transthoracic two-dimensional echocardiography – parasternal long axis view with color flow mapping displaying a mild mitral valve regurgitation. Panel D: Three-dimensional transesophageal view of the mitral valve. Arrow showing vegetation on the posterior mitral leaflet. Panel E: Transesophageal off-axis view of the mitral valve. Arrow showing vegetation on the posterior mitral leaflet. Panel F: Transesophageal off-axis view of the mitral valve with color flow mapping showing a moderate mitral regurgitation (vena contracta width 4.87 mm). Panel G: Transthoracic two-dimensional echocardiography – parasternal short axis view of the mitral valve. Resolution of the vegetation that was detected at the P3 level. Panel H: Transthoracic two-dimensional echocardiography – apical two chamber view. Anterior and posterior mitral leaflets appear intact without vegetation. Panel I: Transthoracic two-dimensional echocardiography – parasternal short axis view of the mitral valve with color flow mapping showing mild residual mitral regurgitation. Abbreviations: AML, anterior mitral leaflet; LA, left atrium; LV, left ventricle; PML, posterior mitral leaflet. P1 (lateral), P2 (middle), and P3 (medial) are the scallops of the posterior mitral leaflet.

A 16-year-old Italian boy was admitted with a seven-day history of persistent fever and chills associated with painful swelling of the right shoulder and pharyngitis. He had previously received four days of cefixime and azithromycin treatment. He did not report any recent dental treatment, surgery, or drug abuse. His past cardiac history was unremarkable. The patient was in good general conditions and had normal vital signs (blood pressure: 125/65 mmHg, regular heart rate: 84 beats/minute). He had a temperature of 100.4°F (38°C). Cardiovascular examination revealed a systolic heart murmur 2/6 at the mesocardium. The pharynx was normal and minimal acne was observed on the skin. Blood tests indicated neutrophilic leukocytosis (white blood cell count: 13x 103/µL; neutrophils: 9.7x103/µL) as well as elevated lactate dehydrogenase (266 mU/mL) and C reactive protein (CRP; 18 mg/dL). The urine analysis was negative for infection. The electrocardiography (ECG) demonstrated sinus tachycardia, and the chest x-ray was normal. A bone marrow aspirate was performed to exclude hematological malignancies. Two sets of blood cultures were drawn, and empirical treatment with amoxicillin/clavulanate plus vancomycin was started. Blood cultures were found to be positive for gram-positive cocci in chains, which were later identified as streptococcus group A (Streptococcus pyogenes). The patient was admitted to the Infectious Disease Department and ceftriaxone (2g, twice daily) plus ampicillin (12g/day) were started. The transesophageal echocardiogram (TEE) documented moderate mitral regurgitation and multiple mobile filamentous structures attached to the posterior mitral leaflet (PML) of 1.1 cm in length suggestive of vegetation (Figure 1A-1I).

The hemodynamic stability of the patient (he had normal values for atrial natriuretic peptide and no signs of heart failure) and the short course of antibiotic treatment were the reasons for not performing urgent surgery on the mitral valve after consultation with cardiac surgeons. Gentamicin (3mg/kg daily) was started instead of ceftriaxone on day seven. His abdominal ultrasonography was negative for liver or splenic embolisms. Painful erythematous nodules (2 to 3mm) were observed on the skin of the right sole, suggestive of secondary lesions. No other skin lesions appeared during the following days, and the neurological examination was steadily normal. The patient underwent weekly ECG evaluations during antibiotic treatment.

A second TEE done on the 12th day of antibiotic therapy found worsening of the endocarditis and growth of the vegetative lesions on the PML. On the 14th day, a cardiac magnetic resonance scan excluded either concomitant rheumatic heart disease or peri-valvular abscess due to Streptococcus pyogenes. At the ECG exams on the 19th and 25th days of hospitalization, the vegetant lesions were not observed (Figure 1 G-I). Gentamicin treatment was continued for two weeks, ampicillin for four weeks, and then the patient was discharged. At the clinical control checkup six months later, the patient was asymptomatic with normal vital signs. A blood test revealed normal white blood cells (WBC) count and CRP levels. Transthoracic echocardiography (TTE) showed a low-grade mitral insufficiency consistent with previous endocarditis of posterior commissure of the mitral valve with normal left ventricular ejection fraction.

## Discussion

Group A β-hemolytic Streptococcus pyogenes colonizes the throat and/or skin, and it is responsible for several purulent infections including pharyngitis, impetigo, necrotizing fasciitis, and streptococcal toxic shock syndrome [1]. Bloodstream infections caused by Streptococcus pyogenes come from a primary source that may be cellulitis, pharyngitis, endometritis, pneumonia, or other localized pyogenic infections [1].

In our case, the source of bacteremia may have been an upper respiratory tract infection, pharyngitis, or skin lesions (acne, folliculitis). Group A Streptococcus pyogenes may induce autoimmune diseases such as acute post-streptococcal glomerulonephritis, rheumatic fever, and rheumatic heart disease; IE has rarely been reported. Based on this, a literature research was performed using PubMed without restriction on search dates. Reports were identified on PubMed using the following keywords: 'infective endocarditis and Streptococcus pyogenes' or 'Streptococcus pyogenes endocarditis'. The literature search was limited to English language articles. Studies not available online were excluded.

Globally, including the present case, 39 cases of endocarditis caused by Streptococcus pyogenes in children and adults have been reported since 1940 [2-14] (Table 1).

**Table 1:** Streptococcus pyogenes endocarditis cases from the literature review Abbreviations: ARDS, acute respiratory distress syndrome; CHF, chronic heart failure; Heart valves: A, aortic; M, mitral; T, tricuspid; n.d., no data.

CASE	YEAR	Age (y)/gender	PRE EXISTING CARDIAC ABNORMALITIES	PRECEDING INFECTION	VALVE AFFECTED	EMBOLI	COMPLICATIONS	THERAPY	SURGERY	OUTCOME
1 [2]	1942	2/M	none	none	M	brain, renal, skin, spleen	splenomegaly, pneumonia	not available	no	death
2 [3]	1961	25/M	none	none	A+M	spleen	kidney abscess	not available	no	death
3 [2]	1975	6 months/M	none	meningitis	A+M	none	aortic failure	penicillin G	no	recovered
4 [2]	1976	14/M	ventricular septal defect, aortic stenosis	tonsillitis	A	none	myocardial abscess	not available	no	death
6 [2]	1980	16/M	none	pharyngitis	A	renal, peripheral	ARDS	not available	no	recovered
7 [2	1984	2/M	pulmonary artery stenosis	pharyngitis	A+M	brain, spleen	cardiac failure	penicillin G; nafcillin and gentamicin	no	death
8 [2]	1988	4/M	none	varicella	A	none	para-aortic abscess	cefuroxime, cefaclor, ampicillin and gentamicin	no	death
9 [2]	1988	4/F	none	none	A	none	polyarthritis	cefotaxime, penicillin G	yes	recovered
10 [4]	1991	63/F	none	pharyngitis	none	none	none	penicillin G and penicillin V	no	recovered
11 [4]	1991	65/M	coronary-artery disease and severe arteriosclerosis	arthritis, cellulitis	M	brain, Janeway lesions	none	penicillin G and penicillin V	no	recovered
12 [4]	1991	69/F	aortic- and pulmonary-valve prostheses	none	none	skin, roth lesion	none	vancomycin and gentamicin	no	recovered
13 [4]	1991	55/M	mitral regurgitation	none	M	none	none	cefazoline, ampicillin, penicillin V	no	recovered
14 [2]	1992	3/F	none	none	M	brain, skin	left-side hemiparesis, pericardial effusion, mitral regurgitation	penicillin G	no	recovered
15 [2]	1994	20/M	none	none	M	none	none	not available	no	death
16 [2]	1998	14/M	none	none	M	brain	cerebral infarction	penicillin, gentamycin	no	recovered
17 [2]	1999	5 months/M	none	varicella	M	skin	respiratory failure, purpura	ceftriaxone and vancomycin, ampicillin, penicillin	yes	recovered
18 [2]	2000	3/M	none	varicella	A	peripheral, skin	joint pain, aortic insufficiency, CHF	penicillin and gentamycin	yes	recovered
19 [2]	2000	4/M	none	none	A	skin	mitral regurgitation, necrosis of toes	not available	yes	recovered
20 [2]	2000	2/M	none	varicella	A	brain, skin	aortic root abscess, aortic valve perforation	not available	yes	recovered
21 [5]	2004	11/M	none	varicella	A	brain, renal, spleen	aortic failure	clarithromycin	yes	recovered
22 [6]	2008	68/M	none	left lower limb weakness	A	none	meningitis, heart-failure, sepsis	ceftriaxone and gentamicin	no	death
23 [7]	2012	71/M	hypertension	none	A, M, T	renal, spleen	aortic regurgitation, coronary artery disease	penicillin G	yes	recovered
24 [2]	2014	6/F	patent foramen ovale, atrial septal defect	erythematous rash, pharyngitis	A, M	skin, brain	aortic root abscess, arrhythmia	ceftriaxone, vancomycin and meropenem, and gentamycin	no	recovered
25 [2]	2014	8 months/F	none	pharyngitis	T	brain	respiratory failure, sepsis, multi-organ failure	penicillin and clindamycin	no	recovered
26 [8]	2010	64/M	none	urinary tract infection pharyngitis	none	none	none	gentamicin, benzylpenicillin	no	recovered
27 [9]	2017	68/M	none	arthritis	none	none	heart and renal failure	ceftriaxone, meropenem, ampicillin and clindamycin, cefazolin	no	recovered
28 [10]	2001	73/F	none	n.d.	A	none	cardiac complications	not available	no	recovered
29 [10]	2005	64/M	none	n.d.	A	none	cardiac complications	not available	yes	recovered
30 [10]	2006	33/M	none	n.d.	T	brain	none	not available	no	recovered
31 [10]	2008	68/F	none	n.d.	M	none	cardiac complications	not available	yes	death
32 [10]	2011	24/M	none	n.d.	none	none	cardiac complications	not available	no	recovered
33 [10]	2013	39/M	none	n.d.	A	yes	cardiac complications	not available	no	death
34 [10]	2013	51/M	none	n.d.	A	none	cardiac complications	not available	yes	recovered
35 [11]	2014	5/M	none	gastroenteritis	M	none	none	amoxicillin and gentamycin	no	recovered
36 [12]	2015	42/M	none	vasculitis arthritis	T	lung, brain	pulmonary thrombosis	ceftriaxone and gentamycin	No	recovered
37 [13]	2017	77/M	biological mitral valve prosthesis, atrial fibrillation, mellitus type 2	otitis media	M	brain	meningitis	ceftriaxone	No	recovered
38 [13]	2017	80/F	none	meningitis	M	n.d.	meningitis	ampicillin and clindamycin	No	recovered

Out of the 39 cases of Streptococcus pyogenes endocarditis identified, 30 were in men (77%). Ages ranged from four months to 80 years with a median age of 32 years. Five patients were intravenous drug users. Pre-existing heart defects were known in 22.2% of the cases. Skin lesions were the predisposing infection in eight cases (20.5%), while pharyngitis was described in seven patients (18%). In four cases (10%), joint pain and arthritis were reported as the first manifestation. Ten cases (26%) had no known preceding infections. Valves on the left side were most commonly involved (88%). Embolic phenomenon occurred in 20 cases (51%) with the central nervous system and skin being the most frequent sites. Ten patients (26%) had cardiac surgery, and one of them died after surgery (10%). The mortality rate was 24% (62.5% before 1990 and 15.4% after 1990) and was mainly due to cardiac failure and/or septic shock; most patients recovered after antibiotic therapy. Compared to Streptococcus pneumonia infectious endocarditis (IE) for which the potential value of valve replacement in preventing early death has been demonstrated, in cases of Streptococcus pyogenes IE, the role for surgery is less mandatory [15]. Most patients treated with antibiotic therapy alone had a favorable outcome (84%). Notably, five of the nine patients that died did so before the 1990s; the microbiological and instrumental techniques from that period are considered obsolete. Based on the limited published data, penicillin G, ampicillin, or ceftriaxone administered intravenously for four weeks are reasonable treatments for Streptococcus pyogenes IE. The addition of gentamicin to penicillin or ceftriaxone for at least the first two weeks of a four-week course of antimicrobial therapy for group B, C, and G streptococcal IE may be considered [16], while another option could be daptomycin plus ampicillin based on the severity of the disease. Vancomycin is reasonable only for patients who are unable to tolerate a beta-lactam antibiotic [16]. In the present case, gentamicin was added to a beta-lactam antibiotic when the echocardiographic images showed the presence of multiple vegetant lesions on the mitral valve.

Streptococcus pyogenes can also produce multiple exotoxins that have the potential to cause end-organ damage or trigger the release of cytokines that can cause tissue injury. Many virulence determinants have been identified using genomic and molecular analyses of Streptococcus pyogenes strains, many of which are related to the processes of adhesion and colonization, innate immune resistance, and the ability to facilitate degradation of tissue barriers and spread in the human body [17]. The key factor of the pathogenicity of Streptococcus pyogenes is the M protein: this surface-exposed antigenic protein is involved in the adhesion of the bacterium to human tissues and in preventing phagocytosis. The Streptococcus pyogenes strain G773 was sequenced in a case of acute endocarditis in a child [11]. This strain possesses a combination of various pathogenetic factors: the M protein is able to bind the C4b-binding protein and immunoglobulin; produces bacteriocins (proteins with bactericidal activity); and has the capacity to colonize the host by eliminating other bacterial species that share the same environment. In seven cases of endocarditis due to Streptococcus pyogenes, fibronectin and fibrinogen-binding genes correlated with the severity of disease manifestations such as an acute onset of illness, large vegetations, the presence of frequent embolic complications, and a high mortality rate. The Streptococcus pyogenes endocarditis strains showed an overrepresentation of several of the fibronectin and fibrinogen binding genes, which probably indicates that the ability to bind the fibronectin is an important step in the development of endovascular streptococcal infections [10].

## Conclusions

When treated with appropriate antimicrobial therapy and supportive therapy promptly, even a severe presentation of Streptococcus pyogenesendocarditis can have excellent outcomes. In our case, antibiotic treatment was associated with clinical recovery. However, because of the relative infrequency of IE caused by Streptococcus pyogenestogether with the lack of studies in children, decisions regarding surgical intervention are best individualized. Epidemiological data and more extensive characterization of virulence factors with a genomic analysis of isolated bacteria and molecular screening are both necessary to further elucidate the pathogenic mechanisms.
